# The Role of FGFR2 as a Novel Biomarker for Treatment of Gastric Cancer—A Literature Review

**DOI:** 10.3390/medicina61111890

**Published:** 2025-10-22

**Authors:** João Lages dos Santos, Rui Caetano Oliveira, João Martins Gama

**Affiliations:** 1 Serviço de Anatomia Patológica, Unidade Local de Saúde de Gaia e Espinho, 4434-502 Porto, Portugal; jpcls117@hotmail.com; 2 Centro de Anatomia Patológica Germano de Sousa, 3030-075 Coimbra, Portugal; 3 Instituto de Histologia e Embriologia, Faculdade de Medicina da Universidade de Coimbra, 3004-528 Coimbra, Portugal; 4 Serviço de Anatomia Patológica, Unidade Local de Saúde de Coimbra, 3004-561 Coimbra, Portugal; joaomartinsgama@gmail.com

**Keywords:** gastric cancer, FGFR2, biomarker

## Abstract

Background: Gastric cancer currently has the third highest mortality rate worldwide among cancer types. Despite gradual declines in mortality rates attributed to improvements in early detection and treatment, outcomes for advanced-stage disease are still poor. The identification of new biomarkers such as fibroblast growth factor receptor 2 (FGFR2) has opened new pathways for directed therapy in gastric cancer. Objective: This review aims to synthesize the current evidence on the role of FGFR2 in gastric cancer, focusing on its biological function and oncogenic mechanisms, diagnostic and prognostic modification, therapeutic targeting, and possible roadblocks in clinical application. Methods: A comprehensive literature search was conducted, selecting studies published between 2015 and 2025 using the MeSH terms “FGFR2 protein, human” [Supplementary Concept]) AND “Stomach Neoplasms”. Articles were screened based on relevance to gastric cancer, language (English), and availability of full text, yielding a final selection of 75 studies, including preclinical research, clinical trials, and reviews. Findings: We compiled and reported the evidence on FGFR2 detection methods, intra-tumoral heterogeneity of FGFR2 expression, effects of FGFR2 expression on prognosis, current therapy options targeting FGFR2, and challenges in pursuing this modality of treatment. Conclusion: FGFR2 represents a promising biomarker and therapeutic target in gastric cancer.

## 1. Introduction

As per the latest data from 2024, GLOBOCAN reported that gastric cancer ranks as the fifth most common cancer type worldwide and the fourth most lethal cancer type in terms of cancer-related deaths [1,2]. Recent data shows an improvement in 5-year survival rates, which can be attributed to a combination of standardized surgical techniques, improvements in perioperative management and targeted therapy such as immunotherapy or human epidermal growth factor receptor 2 (HER2) blocking [3].

Biomarkers such as microsatellite instability (MSI), HER2, and programmed death-ligand 1 (PD-L1) have already made an impact in the treatment of gastric cancer, and new biomarkers have recently been identified, such as claudin 18.2 (CLDN18.2) and fibroblast growth receptor 2 (FGFR2) [4,5].

HER2-targeted therapy with trastuzumab plus chemotherapy in advanced HER2-positive gastric cancer has improved median overall survival (OS) from 11.1 to 13.8 months (hazard ratio 0.74, 95% CI 0.60–0.91), reaching 16.0 vs. 11.8 months when in the most strongly HER2-positive subgroup (immunohistochemistry (IHC) 3+ or IHC 2+ with fluorescence in situ hybridization (FISH) positivity) compared to conventional chemotherapy regimens [6]. In a similar manner, tumors with MSI have also been identified as having more favorable response to treatment with immune-checkpoint blockers such as anti-PD-L1 drugs, as these tumors have a high mutation burden, leading to an abundance of epitopes, making them more immunogenic. This has led to survival gains in the treatment of gastric cancer, both with pembrolizumab alone and with pembrolizumab in combination with chemotherapy [7].

CLDN18.2, a tight-junction protein highly expressed in gastric epithelial cells, has recently emerged as a promising targetable biomarker for gastric cancer, with promising survival benefits in recent phase III trial results using Zolbetuximab, an anti-CLDN18.2 antibody [8].

Despite the development of new treatment options, a significant number of patients lack these targetable biomarkers: the frequency of HER2 expression in gastric cancer is heterogeneous, ranging from 4.4% to 53.4%, with the mean being 17.9% [9]; only 10 to 22% of gastric tumors present MSI [10]; and although PD-L1 positivity in gastric cancer at a cutoff of CPS ≥ 1 has been frequently observed in clinical trials, ranging from 60.4% to 85.1% [11], the clinical benefits of immunotherapy are more significant in patients with higher CPS scores [12]. Additionally, these tumors can present intra-tumor heterogeneity, which can impair treatment response, develop resistance to treatment, or develop mutations that produce a negative effect on survival. As an added challenge, the possibility for combined treatment is limited, since HER2 amplification does not correlate with PD-L1 expression of MSI status [13].

These limitations make clear the need for new molecular targets such as FGFR2. In this review, we aim to explore the effects of FGFR2 overexpression in gastric cancer, the utility of targeting FGFR2 in the treatment of gastric cancer, and the challenges in using this biomarker.

## 2. Methods

### 2.1. Objectives

FGFR2 has been identified as a promising biomarker with prognostic implications for gastric cancer and a potential molecular target for personalized medicine. In this narrative literature review, we aim to review and summarize the most relevant articles published in the past decade on the role of FGFR2 in gastric cancer, the effect of overexpression of FGFR2 on the prognosis, and current efforts in developing targeted therapeutic options.

### 2.2. Literature Search Strategy

To identify research articles relevant to the role of FGFR2 in gastric cancer, an initial extensive search of the available literature was conducted, with PubMed as the primary database, using the MeSH terms “FGFR2 protein, human” [Supplementary Concept] AND “Stomach Neoplasms” [Mesh]. To enhance comprehensiveness, additional searches were conducted using Google Scholar, Europe PMC, MedRxiv, and BioRxiv using the same terms, as well as combinations of “FGFR2”, “gastric cancer”, “clinical trial, “Phase I trial”, and “Phase II trial”. Supplementary targeted searches were performed for specific clinical trials on ClinicalTrials.gov. The first search was performed in March 2025, and the final search was realized in September after the first round of peer reviews.

For information regarding ongoing or completed clinical trials, we consulted ClinicalTrials.gov.

References were managed in Mendeley Reference Manager (Version 2.138.0) to avoid duplication.

### 2.3. Eligibility Criteria

The inclusion and exclusion criteria used for the selection of articles were as follows:

### 2.4. Inclusion Criteria

- Studies published between January 2015 and April 2025.
- Studies published in English.
- Studies investigating FGFR2 expression, amplification, signaling pathways, therapeutic targeting, or clinical implications with relevance to gastric cancer.
- Preclinical studies (in vitro and in vivo models), clinical trials, case reports, and meta-analyses relevant to FGFR2 in gastric cancer.
- Review articles providing comprehensive insights into FGFR2’s role in gastric cancer.

### 2.5. Exclusion Criteria

- Studies not published in English
- Commentaries or editorials without significant primary data.
- Studies focused on FGFR2 in other malignancies without relevance to gastric cancer.

**Figure 1:**
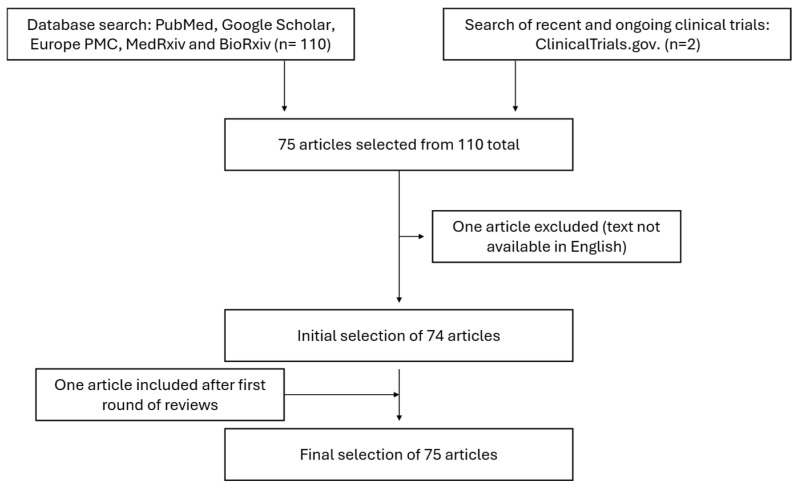
Flowchart representing article selection process.

The initial search process yielded 110 potentially relevant articles. After selecting the resulting articles for relevance and originality, our initial selection was a total of 75 articles. One article was removed due to being published only in Mandarin, and one article was added after the first round of reviews. The methodology can be seen in Figure 1.

The selected studies were then grouped according to the topics approached:

1. Detection of FGFR2 amplification or mutations.
2. Tumor heterogeneity of FGFR2 expression.
3. Effect of FGFR2 on prognosis.
4. Directed therapy targeting FGFR2.
5. Resistance mechanisms and other challenges in targeted FGFR2 therapy.

## 3. Findings and Discussion from the Last Decade

### 3.1. FGFR2

FGFR2 is a single-pass transmembrane receptor tyrosine kinase of the FGFR family, which includes FGFR1 through FGFR4, that is involved in the mitogen-activated protein kinase (MAPK) and AKT pathways. These receptors mediate cellular responses to fibroblast growth factors (FGFs), regulating key biological processes such as proliferation, differentiation, angiogenesis, and survival [14,15]. Receptors of the FGF family share a common overall structure: they consist of an extracellular component of three immunoglobulin-like domains (D1–D3), with an acid box region between Ig1 and Ig3 and the ligand binding domain between D1 and D3; a transmembrane component that anchors the receptor in the cell membrane; and an intracellular domain with tyrosine kinase activity [14,15].

Upon binding the respective FGF ligand, FGFR2 undergoes dimerization and autophosphorylation, activating the intracellular tyrosine kinase domain and triggering downstream signaling cascades such as the MAPK, PI3K-AKT, PLCγ, and STAT pathways. Upon phosphorylation, FRS2 recruits GRB2, which binds SOS and GAB1. SOS activates the RAS-MAPK pathway, while GAB1 activates phosphatidylinositol (PI)-3 kinase. FGFRs also activate the STAT pathway and phosphorylate PLCγ, which, in turn, generate arachidonic acid and elevate intracellular calcium levels, activating calcium-dependent protein kinase C (PKCs) [14].

Ligand-receptor specificity for FGFR is modulated by alternative splicing of the FGFR2 gene, which consists of over 21 exons, resulting in the production of multiple isoforms of FGFR2 with functional differences, of which FGFR2b and FGFR2c are distinct examples: FGFR2b is preferentially expressed in epithelial cells and preferentially binds epithelial FGFs such as FGF7 and FGF10, while FGFR2c is predominantly expressed in mesenchymal cells and preferentially binds mesenchymal FGFs such as FGF2 and FGF4. These isoforms are pathologically implicated, as during epithelial–mesenchymal transition, a switch to expression of the FGFR2c isoform favors cell motility and invasion [16,17].

**Figure 2:**
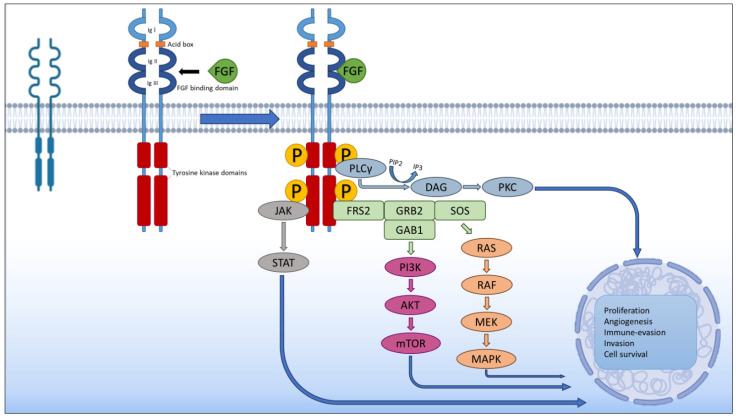
Diagram of FGFR signaling pathways. FGF-FGFR binding induces receptor dimerization, leading to phosphorylation of FRS2, PLCγ, and JAK. This activates four major signaling cascades: (1) FRS2 recruits GRB2 and SOS to initiate RAS–MAPK signaling; (2) GRB2–GAB1 activates the PI3K–AKT–mTOR pathway; (3) PLCγ hydrolyzes PIP2 to generate IP3 and DAG, leading to PKC activation; and (4) JAK–STAT signaling. Figure created with BioRender™.

FGFR2 also presents slower internalization after ligand binding when compared to FGFR1 and FGFR3, which leads to prolonged signaling, contributing to the oncogenic potential of this receptor [14,18]. A schematic representation of the FGFR signaling pathways can be seen in Figure 2.

### 3.2. FGFR2 in Cancer

As FGFR2 is implicated in signaling pathways that promote cell proliferation, inhibition of apoptosis, and angiogenesis, it has been identified as an oncogene with potential for directed therapy [17,19,20,21].

FGFR2 can act as a molecular driver of cancer through several different mechanisms. Gene amplification of FGFR2 has been identified in gastric and breast cancers, leading to overexpression of the protein and abnormally high receptor density at the cell membrane [22,23]. Fusion genes involving FGFR2, such as FGFR2-ACSL5, create permanently active chimeric proteins and have been identified in cholangiocarcinoma and breast and lung cancers [24]. Multiple mutations of FGFR2, both germinal and somatic, have been identified in several types of cancer. Germinal missense mutations of the FGFR2 gene are present in several congenital skeletal disorders, including Apert syndrome, Beare–Stevenson syndrome, Crouzon syndrome, Jackson–Weiss syndrome, and Pfeiffer syndrome [17]. Somatic missense mutations of FGFR2 can lead to ligand-independent activation of FGFR2, and multiple mutations have been identified as playing a driving role in breast, endometrial, and lung cancers [14,17].

## 4. Detection of FGFR2 Amplification or Mutations

### 4.1. Detection Methodology

**Figure 3:**
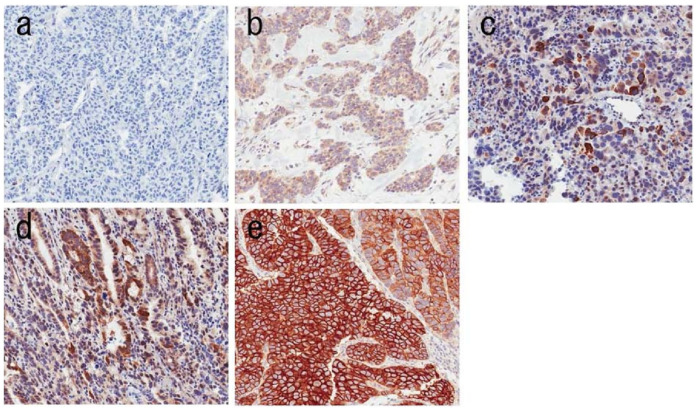
Examples of FGFR2b immunohistochemistry staining at 200× magnification. Images (**a**–**e**) show IHC score expressions of 0, 1, 2, 3, and 4, respectively. Scores are defined as follows: 1—<10% of tumor cells staining weakly; 2—≥10% of tumor cells staining weakly; 3—≥10% but <50% of tumor cells staining strongly; 4—≥50% of cells staining strongly. Original picture available in DOI: https://doi.org/10.1093/jjco/hyab104 (last accessed on 29 September 2025) [26]; reproduction allowed by License CC BY-NC 4.0.

In the past decade, multiple different detection methods for FGFR2b amplification have been compared across multiple sample types. FISH is considered to be the gold standard to detect FGFR2 amplification, and IHC seems to correlate well with FISH results: a comprehensive comparison for detection of FGFR2 amplification was conducted in frozen, biopsy, and surgical specimens, evaluating both copy numbers through polymerase chain reaction (qPCR) and IHC and correlating the results of all specimens with FISH (examples of IHC scoring can be observed in Figure 3; FGFR2b expression was semiquantified in the tumor area using a microscope with a grid scale, and heterogeneous expression was defined as positivity for FGFR2b in less than 50% of the tumor area).

The results of this study identified frozen specimens as the most sensitive for FGFR2 amplification and validated the use of biopsy specimens for FGFR2 testing, as these were equivalent to or better than surgical specimens since both qPCR and IHC showed excellent correlation with FISH results. mRNA levels were found to not always correspond with amplification status [25].

Alternative techniques to FISH have been used for spatial detection of FGFR2 amplification, such as RNA in situ hybridization for FGFR2 mRNA alongside gene copy assessment via dual-color in situ hybridization (DISH). This combined approach identified a correlation between FGFR2 mRNA expression and gene amplification in a cohort of 1036 gastric cancer patients. The use of techniques with spatial resolution allowed for the localization of FGFR2 expression within histologic substructures and identified cases with intra-tumoral heterogeneity of FGFR2 expression [27].

More recently, liquid biopsy approaches have also been tested as methods of noninvasive genomic analysis, particularly circulating tumor DNA (ctDNA). Patients with FGFR2-positive circulating tumor cells (CTCs) had significantly worse recurrence-free survival, and there was correlation between CTC and primary tumor FGFR2 expression [28]. Two studies detected FGFR2 amplifications by ctDNA sequencing that were not detected in the corresponding tissue biopsies or had subclonal or heterogeneous amplification. Furthermore, patients with FGFR2+ ctDNA were observed to have poorer survival but responded to FGFR inhibitors, even in cases with negative tissue biopsies [29,30].

The use of a novel FGFR2-targeting peptide for early detection of FGFR2 overexpression in early-onset gastric cancer and esophageal cancer was investigated, resorting to phage display and fluorescence to identify FGFR2 cell surface overexpression, and finding significantly greater binding in esophageal specimens with high-grade dysplasia compared to Barret’s esophagus or normal mucosa, as well as significantly greater binding in specimens of esophageal squamous cell carcinoma and gastric cancer compared to normal mucosa [31].

### 4.2. Prevalence of FGFR2 Amplification and Overexpression

The reported prevalences of FGFR2 amplification and overexpression in gastric tumors vary: amplification was observed in 11.5% (7/61) of cases in one cohort [32], while overexpression of FGFR2 has been observed in 16.2% (612/3782) of cases (20), going as high as 42% (73/173) in one cohort [20,26,33].

The prevalence and prognostic significance of FGFR2 amplification were described in 2015 in a cohort of 61 patients with advanced-stage gastric cancer undergoing treatment with chemotherapy. FGFR2 amplification was detected using FISH in 11.5% of cases, and these patients had significantly shorter overall survival compared to those with no FGFR2 alteration [32].

A study comprising over 5557 solid tumors of Chinese patients used a panel-based NGS assay to report FGFR1–4 alterations, including amplifications, mutations, and rearrangements, across a wide range of cancers. Of all tumors with identified FGFR alterations, gastric tumors registered as the fourth highest at 12.2% (31/254), with 7.9% (20/254) of those having FGFR2 alterations [34].

The prevalence of FGFR2b protein overexpression, evaluated through IHC, was described in the prescreening phase of the FORTITUDE-101 trial. In this sample, 37.8% (1430/3782) of tumors exhibited any % of FGFR2b positivity in tumor cells, and 16.2% (613/3782) of tumors had FGFR2b overexpression (defined as >10% of tumor cells staining positive), fulfilling the criteria for trial eligibility [20].

### 4.3. Concurrent Markers and Co-Expression Studies

Co-expression of FGFR2 and other relevant biomarkers in gastric cancer, such as HER2, EGFR, MET, and Claudin 18.2, has also been a topic of interest. Overexpression of FGFR2 alone has been linked to worse outcomes, and tumors expressing multiple biomarkers, as is the case for triple-positive tumors (FGFR2+, HER2+, c-MET+) have been associated with having the worst prognosis in some cohorts [35]. In a cohort of 950 patients with treatment-naïve gastric adenocarcinoma, 63.1% of tumors expressed at least one receptor tyrosine kinase (HER2, EGFR, MET, FGFR2), while 22.7% of these simultaneously expressed multiple biomarkers [36].

Despite carrying worse implications for prognosis, co-expression was observed only in a subset of cases: one study investigating co-expression of MET and FGFR2 in metastatic gastric cancer found MET-FGFR2 amplification in only 0.6% of their cohort [37], while another study in a cohort of 1000 patients found that FGFR2 amplification was associated with CLDN18.2 expression in 44.2% of FGFR2-positive cases [8].

### 4.4. Molecular Mechanisms of FGFR2 Regulation

Multiple molecular mechanisms have been identified for regulating FGFR2 expression.

A relationship of reciprocal regulation has been identified in vitro between CD44 and FGFR2 via c-Myc. CD44 knockdown reduced FGFR2 expression and impaired tumor formation, and the reverse was also observed, as FGFR2 knockdown reduced CD44 levels and stem cell markers, paradoxically increasing c-Myc and SOX-2 [38].

RNA helicase DDX6 was also identified as playing a role in the post-transcriptional regulation of both FGFR2 and HER2 in gastric cancer cells. DDX6 overexpression was observed in 60% of gastric cancer samples and 83% of FGFR2-positive cases, and DDX6 knockdown reduced FGFR2 and HER2 protein expression without affecting mRNA levels [39].

A co-expression study evaluated the expressions of HER2, EGFR, MET, and FGFR2 using tissue microarrays (TMAs), immunohistochemistry (IHC), and FISH of 950 patients with gastric adenocarcinoma, detecting FGFR2 positivity in 31% of cases using TMAs but with most of the FGFR2-positive tumors only showing strong staining in IHC in between 10 and 30% of the IHC staining area. Interestingly, the authors note that although both nuclear and cytoplasmic expression of FGFR2 are considered as features of “FGFR2-enriched gastric cancer”, there are both clinical and IHC staining differences between nuclear and cytoplasmic FGFR2: while the authors consider that both nuclear and cytoplasmic overexpression of FGFR2 seem to be associated with a worse prognosis, with other authors highlighting nuclear overexpression as the worse of the two, antibodies targeting FGFR2 did not seem to attack nuclear FGFR2 [36].

Epigenetic regulation of FGFR2 has also been studied, with FGFR2 promoter hypomethylation highlighted as a promising non-invasive biomarker for detecting gastric cancer and intestinal metaplasia: FGFR2 promoter methylation levels measured with MSRE-qPCR differ sharply between control groups (approximately 93% methylation levels), intestinal metaplasia groups (approximately 70% methylation levels), and gastric cancer groups (26 to 28% methylation levels). This test was 100% sensitive and 100% specific when comparing gastric cancer to healthy controls with a methylation cut-off of <74.25% and was 85% sensitive and 80% specific when comparing gastric cancer to intestinal metaplasia, with the methylation cut-off defined as 44.45% [40].

Amplification and demethylation of FGFR2 was also identified as increasing ESRP1 expression and leading to the preferential expression of FGFR2-IIIb. ESRP1 expression was inversely correlated with the expression of FGFR2-IIIc, an alternative FGFR2 splicing isoform. On survival analysis, patients with diffuse-type tumors with low-FGFR2-IIIc had better overall survival than patients with FGFR2-IIIc high-expressing diffuse tumors. As the IIIc isoform is also expressed in mesenchymal cells, the preferential expression of FGFR2-IIIc seems to correlate with a more aggressive phenotype, serving as evidence of the epithelial–mesenchymal transition [41,42].

A table summarizing the data for each detection method is available below (Table 1).

## 5. Tumor Heterogeneity of FGFR2b Expression

### 5.1. Intra-Tumoral Heterogeneity of FGFR2 Expression

FGFR2b expression is highly heterogenous, with up to 93% of cases showing heterogeneous expression in some cohorts [37,43,44], leading to false-negative rates of 12.2% with three biopsies. Increasing the sampling to six biopsies reduced false-negative risk to near 0% [45]. Intra-tumoral heterogeneity was also observed by comparing FGFR2b expression in biopsy with the corresponding surgical specimen, as one case classified by FISH as FGFR2-negative in the biopsy sample was later confirmed to have FGFR2 overexpression in the surgical specimen. Fortunately, in this cohort, this single discordant case did not change the equivalence or superiority of the biopsy specimen vs. the surgical specimen [25].

Although tumor heterogeneity of FGFR2 expression is highly prevalent and presents a limitation for screening accuracy [20], it can potentially be overcome using ctDNA sequencing in liquid biopsy, as this technique successfully identified FGFR2-positive cases that were negative on biopsy (7.7% (28/365) with ctDNA sequencing vs. 2.6%, 3.4%, and 4.4% with tissue analysis, as described in the publicly available tissue-based databases: the GI-SCREEN studies, The Cancer Genome Atlas (TCGA), and Memorial Sloan Kettering Cancer Center (MSKCC) [29]. FGFR2-positive circulating tumor cells were significantly correlated with FGFR2 overexpression and gene amplification in the surgical specimen of a gastric tumor, which validates a potential liquid-biopsy-based approach towards screening and monitoring for FGFR2-amplified gastric cancer [28].

### 5.2. Molecular and Genetic Heterogeneity

FGFR2b overexpression in gastric cancer can originate from different mechanisms. Molecular heterogeneity refers to differences in the pathways regulating FGFR2 expression (as mentioned previously: isoform switching, epigenetic regulation, interaction with co-regulators), while genetic heterogeneity implies alterations at the DNA level (such as amplification, mutations, and fusion genes). Despite being less frequently observed than gene amplification, several FGFR2 fusion genes have been identified in gastric cancer as well as in common bile duct cancer, hepatocellular carcinoma, pancreatic cancer, and sarcomas. Some of the observed fusion partners for FGFR2 in gastric cancer include *TACC2*, *BICC1*, *BTBD16*, *WAC*, *HFM1*, *HOOK1*, *INPP5F*, *C10orf90*, *WDR11*, *APIP*, and *CD44.* FGFR2 gene amplification and fusion can also be concurrent, occurring most frequently in gastric cancer [46,47].

## 6. Prognostic Significance of FGFR2 Expression

### 6.1. FGFR2b Overexpression

FGFR2b overexpression is consistently identified as an independent predictor of poor prognosis in patients with gastric cancer [23,32]. These patients present lower overall survival rates, lower progression-free survival, and higher recurrence risks and more frequently have poorly differentiated adenocarcinoma, lymph node metastasis, peritoneal seeding, and distant metastasis, as FGFR2 signaling promotes migration and invasion in gastric cancer cells, as well as promoting an angiogenic tumor microenvironment [23,26,48,49,50,51,52,53,54,55,56,57,58,59].

### 6.2. FGFR2b Expression and Chemotherapy Response

FGFR2b expression has been identified as having a negative effect on chemotherapy response, as patients with FGFR2-positive tumors present lower overall survival while undergoing adjuvant or palliative treatment [60]. This effect on treatment response was observed for fluoropyrimidine regimens, platinum-based regimens, and, interestingly, in response to trastuzumab for HER2-positive GC: in a cohort of patients with gastric cancer with HER2 expression, patients whose tumors presented FGFR2 overexpression had worse response to trastuzumab compared to the FGFR2-negative group [32,60,61,62,63].

This effect of FGFR2b overexpression on resistance to directed therapy to other biomarkers was also observed in response to MET inhibitors. A study using patient-derived MET-amplified gastric cancer xenografts demonstrated that these tumors did not respond to a selective MET inhibitor but responded to combined treatment with MET and FGFR2 inhibitors, which, on additional analysis, highlighted the contribution of FGFR2 overexpression to treatment resistance [64].

## 7. Directed Therapy Targeting FGFR2

### 7.1. FGFR2-Targeted Tyrosine Kinase Inhibitors (TKIs)

Several TKIs have been tested for directed therapy against FGFR2. These small molecules enter the cell and bind to the intracellular tyrosine kinase domain of FGFR2, preventing phosphorylation and downstream signaling [65].

#### 7.1.1. AZD4547

AZD4547 is a selective inhibitor of FGFR2 that demonstrated a significant reduction in GC cell proliferation and induced apoptosis and G1 cell cycle arrest in patient-derived xenografts of FGFR2-amplified cell lines [65]. A translational clinical trial demonstrated that tumors with high-level clonal FGFR2 gene amplification showed response to treatment (four out of nine patients with FGFR2-amplified gastroesophageal cancer), although the sample size for the gastric cancer cohort was limited [66]. The SHINE study (NCT01457846), a comparative randomized, open-label, phase II study of the efficacy and safety of AZD4547 versus paclitaxel for advanced gastric adenocarcinoma with FGFR2 polysomy or gene amplification, showed no significant benefits in progression-free survival (PFS) for AZD4547 monotherapy. Despite this, the drug was well tolerated [67].

#### 7.1.2. Futibatinib

Futibatinib is an irreversible FGFR1-4 inhibitor that was first described in 2020, demonstrating potent tumor growth inhibition in multiple preclinical cancer models with FGFR2 expression [68]. Phase I clinical trials (NCT02052778; JapicCTI-142552) identified hyperphosphatemia as the only dose-limiting toxicity (DLT) observed, and no maximum tolerated dose was reached, suggesting broad safety at therapeutic doses [69]. A phase II clinical trial (NCT04189445) demonstrated tumor shrinkage in 58% of patients with FGFR2-amplified gastric cancer, but the objective response rate (ORR) varied between 17,9% and 26%. Patients with higher copy numbers of FGFR2 had better response to Futibatinib, and the most common adverse effects were hyperphosphatemia, fatigue, and decreased appetite [70,71].

Two patients with high-level FGFR2-amplified metastatic gastric cancer were successfully treated with futibatinib after progression on multiple lines of chemotherapy, with significant tumor shrinkage, symptom relief, and reduced tumor marker levels. However, one patient developed rapid progression after five months, possibly due to acquired resistance [72].

#### 7.1.3. KIN-3248

KIN-3248 is a selective, irreversible, orally bioavailable inhibitor of FGFR1-4. A phase I trial (NCT05242822) in patients with advanced solid tumors with FGFR2 gene alterations aimed to determine the maximum tolerated dose and antitumor activity but was terminated early due to commercial concerns. Despite the early termination, 5 out of 54 patients (9.26%) exhibited partial response, and only one DLT (hypersensitivity) occurred, with the most common adverse events being hyperphosphatemia, diarrhea, and stomatitis [73].

#### 7.1.4. Infigratinib

Infigratinib, an FGFR1-3 inhibitor that was approved by the FDA in May 2021 as second line for advanced cholangiocarcinoma with FGFR2 fusion genes but later revoked in 2024, has been tested for locally advanced or metastatic gastric cancer or gastroesophageal junction adenocarcinoma with FGFR2 gene amplification in the LB1001-201 trial (NCT05019794), a single-arm phase II trial with two cohorts, with an ORR of 25% and a disease control rate (DCR) of 80%, with 15 out of 19 patients presenting tumor shrinkage, the maximum from baseline being a reduction of 78.5% [74,75].

#### 7.1.5. Pemigatinib

Pemigatinib, a potent FGFR1–4 inhibitor approved by the FDA and EMA for second-line treatment of advanced cholangiocarcinoma with FGFR2 mutations, was used to treat a case of FGFR2-altered advanced gastric cancer after exhausting multiple prior treatment options, among which were docetaxel, cisplatin, irinotecan, and nivolumab. Comprehensive genomic profiling revealed both FGFR2 amplification and rearrangement, and off-label use of pemigatinib resulted in rapid reductions in tumor markers (CEA and CA19-9) and notable clinical improvement through five treatment cycles (approximately three months) until progression was observed both by imaging and elevation of tumor markers shortly after the fifth cycle [76].

### 7.2. FGFR2-Targeting Antibodies and Antibody–Drug Conjugates

#### Bemarituzumab

Bemarituzumab is a humanized monoclonal antibody that selectively targets the third immunoglobulin domain responsible for ligand specificity of FGFR2, inhibiting downstream phosphorylation. It is currently the only FGFR2-targeting drug with Breakthrough Designation by the FDA for frontline treatment of FGFR2b-overexpressing (defined as >10% of tumor cells staining positive for FGFR2b IHC) metastatic and locally advanced gastric and gastroesophageal adenocarcinoma [77].

Having previously demonstrated safety and improved observable response rates when compared to immunotherapy in phase I studies [78], the FIGHT trial (NCT03694522), a global, double-blinded, randomized, and placebo-controlled phase II trial, was conducted. Patients were assigned to two arms, bemarituzumab+mFOLFOX6 (*n* = 77) or placebo-mFOLFOX6 (*n* = 78), and the primary endpoint was PFS. The secondary endpoints were OS, ORR, and safety. After a minimum follow-up of 24 months (median 13.5 months), the combined therapy with bemarituzumab-mFOLFOX6 produced a longer median PFS than placebo-mFOLFOX6 (9.5 vs. 7.4 months), with a median OS of 19.2 vs. 13.5 months. ORR was similarly higher for the bemarituzumab arm (48.1 vs. 33%) [79,80,81].

Following based on the FIGHT trial, as of July 2025, the FORTITUDE-101 trial (NCT05052801), a randomized, multi-center, double-blind, placebo-controlled phase 3 clinical trial comparing bemarituzumab+mFOLFOX6 versus placebo+mFOLFOX6 in patients with treatment-naïve advanced gastric or gastroesophageal junction cancer with FGFR2b overexpression, is ongoing, with the main objective of determining efficacy. The study began in March of 2023 and currently has 547 patients enrolled, with estimated completion by June 2026, having achieved primary completion in June 2025. Table 2 provides a summary of some clinical trials.

## 8. Challenges in Targeted FGFR2 Therapy

### Resistance to FGFR Inhibitors

A recurrent challenge in studies on drugs targeting FGFR2 is the development of treatment resistance, leading to disease progression. Several mechanisms have been studied using patient-derived models of FGFR2-amplified gastric cancer. A study showed that prolonged AZD4547 treatment could lead to resistance without gatekeeper mutations or reactivation of MAPK/AKT signaling by resorting to a protein kinase C-mediated, AKT-independent pathway. This alternative signaling pathway led to preservation of pro-survival proteins and maintained tumor viability. Inhibition of PKC restored GSK3β activity and partially resensitized resistant tumors to FGFR2 inhibition [82].

Alternative pathways to FGFR2 signaling were also observed in a kinome-wide CRISPR/Cas9 loss-of-function screen in FGFR2-amplified KatoIII cells treated with AZD4547, with ILK loss markedly enhancing FGFR inhibitor efficacy and having downstream effects on GSK3β phosphorylation, while EGFR/HER2 signaling could partially compensate for FGFR blockade. Combined use of ILK or EGFR/HER2 inhibitors with AZD4547 improved sensitivity to FGFR inhibition [83]. Other studies observed development of resistance through a JHDM1D-BRAF fusion gene, which promoted constitutive BRAF dimerization, leading to MAPK pathway activation independent of FGFR2. The resulting clones were insensitive to FGFR inhibition but highly sensitive to RAF dimer inhibitors and MEK inhibition [84].

Tumor microenvironment adaptations also present a challenge to target FGFR2, as diffuse-type gastric cancer cells can switch their driver pathways from FGFR2b expression to CXCR4 under hypoxia via SDF1 produced by cancer associated fibroblasts [85].

Combined treatment with FGFR inhibitors and inhibitors of secondary pathways seems a promising route towards overcoming tumor resistance, but this requires clinical validation and raises safety concerns.

## 9. Practical Approach

In this section, we aim to consolidate the information presented thus far as a practical guide to the evaluation of FGFR2b-positive GC.

**Specimen collection**

Biopsy specimens should be the first medium to test for FGFR2 expression, with at least six samples recommended so as to minimize false negatives due to tumor heterogeneity [25]. Despite frozen tissue yielding higher DNA quality, FFPE is adequate for both IHC and molecular assays, so long as fixation and processing are optimized to preserve antigen availability [86].

**IHC**

IHC scoring of FGFR2b expression is scored from 0 to 4, with each score defined as follows [26]:

1. <10% of tumor cells staining weakly;
2. ≥10% of tumor cells staining weakly;
3. ≥10% but <50% of tumor cells staining strongly;
4. ≥50% of cells staining strongly.

As bemarituzumab is currently the most promising direct therapy for FGFR2-overexpressed GC, we restate that, as part of the inclusion criteria defined in the FIGHT (NCT03694522) [79] and FORTITUDE-101 (NCT05052801) (20) trials, FGFR2 overexpression by IHC is defined as moderate-to-strong (2+/3+) membranous staining in ≥10% of tumor cells.

Equivocal IHC cases are defined as cases with 5–10% of positive tumor cells, weak or moderate staining intensity, or ambiguous/heterogeneous staining patterns that do not clearly fit the criteria for positivity or negativity [25,53].

**FISH and RNA ISH**

Reflex confirmatory testing should be considered for negative or equivocal IHC cases or cases with a high clinical suspicion of FGFR2 amplification. FISH is the gold standard for detecting FGFR2 amplification, and an FGFR2/CEN10 ratio ≥ 2 should be considered positive [25,27].

RNA ISH is also validated, with mRNA overexpression defined as a score of ≥2 [27].

**Liquid biopsy**

If tissue-based testing is negative but clinical suspicion remains high (i.e., disease progression, limited biopsy material, or suspected sampling error), liquid biopsy can be used to detect FGFR2 overexpression. CtDNA analysis can be used and is considered positive with a fold-change threshold of ≥1.4 [29,30]. CTC assays are also a valid approach, with positivity defined with ≥5 FGFR2-positive cells per 10 mL of blood [28].

**Figure 4:**
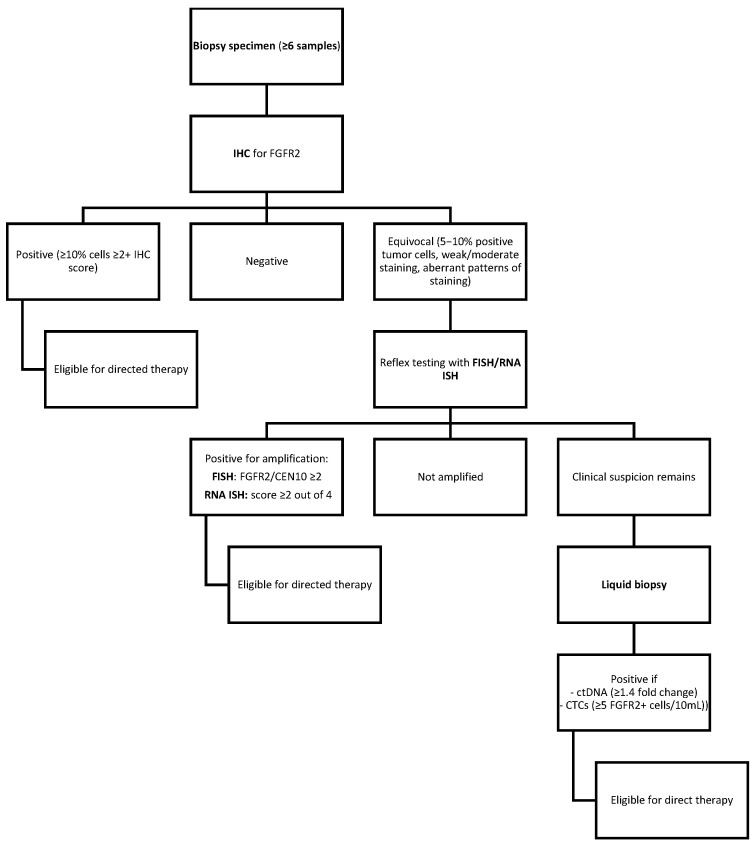
Decision-tree flowchart for FGFR2 testing.

This information is presented below in a decision-tree format (Figure 4).

We include a suggestion for a structured report of FGFR2b IHC scoring.

Example of structured pathology report for FGFR2b IHC:

- Specimen type: Biopsy (six samples).
- Assay performed: Immunohistochemistry (IHC) for FGFR2b.
- Percentage of positive tumor cells: 25%.
- Staining intensity/score: 3+ (strong membranous staining in ≥10% but <50% of tumor cells or ≥50% weak–moderate staining).
- Staining distribution: Diffuse, strong.
- Nuclear/cytoplasmic staining: Predominantly cytoplasmic (membranous staining also observed).
- Interpretation/Result: Positive (3+).

## 10. Conclusions

FGFR2 has emerged as both a promising biomarker with prognostic significance and a potential therapeutic target in gastric cancer. Amplification and overexpression of FGFR2 can be availably detected in biopsy through IHC but also in liquid biopsy and surgical specimens, and they are consistently associated with aggressive tumor behavior, lower survival, and resistance to current therapy options, opening the way for directed treatment.

In the past decade, multiple approaches have been explored to target FGFR2, including tyrosine kinase inhibitors and monoclonal antibodies. Although TKIs such as AZD4547 demonstrated efficacy, clinical benefits were limited. More recent agents, such as futibatinib, have shown more encouraging efficacy, although there are still challenges regarding toxicity profiles and acquired resistance. The most promising directed treatment option is currently bemarituzumab, a selective anti-FGFR2b antibody that has demonstrated favorable safety and efficacy when used in a combined regimen with mFOLFOX6 and that is currently ongoing validation in a phase III clinical trial.

Despite these advancements, the small prevalence of FGFR2 amplification and overexpression, tumor heterogeneity, and the emergence of resistance mechanisms represent challenges in the directed treatment of these tumors.

As of today, FGFR2 presents as a relevant new biomarker with prognostic significance that stands to be integrated into current diagnostic and treatment algorithms, as the advancement of these new treatment modalities may significantly improve outcomes for this subset of patients with aggressive gastric cancer.

## Figures and Tables

**Table 1:** Summary of each detection method for FGFR2 amplification.

Test Platform	Positivity Thresholds	Specimen	Clinical Endpoint Reported	Notes and References
**FISH (fluorescence in situ hybridization)**	Amplification defined as FGFR2/centromere 10 (CEN10) ratio > 2.10	Biopsy, frozen, surgical specimens	Correlated with IHC and qPCR results; prognostic association with shorter OS in some cohorts	Park 2015 [25] showed biopsy equivalent/superior to surgical specimen. False negatives possible in heterogeneous tumors.
**IHC (immunohistochemistry)**	Scores defined as: 1- <10% of tumor cells staining weakly; 2- ≥10% of tumor cells staining weakly; 3- ≥10% but <50% of tumor cells staining strongly; 4- ≥50% of cells staining strongly	Biopsy, surgical specimens	Associated with amplification (validated vs. FISH); stronger positivity associated with poorer outcomes	Park 2015 [25]: strong correlation with FISH. Rha 2025 [20]: 16.2% >10% positive tumor cells → trial eligibility. Heterogeneity common (often <50% area staining).
**qPCR**	≥8 FGFR2 copies correlated best with FISH (κ = 1.0, *p* < 0.001) ROC curve analysis found the optimal cutoff to be >6.71	Frozen vs. FFPE tissue	Frozen DNA higher-quality; FFPE more degraded	Park 2015 [25]: Frozen > FFPE for sensitivity; biopsy > surgical tissue for DNA quality.
**DISH (dual-color in situ hybridization)**	FGFR2/CEN10 ≥ 2	Tissue sections	Correlation between FGFR2 mRNA expression and gene amplification	Kuboki 2018 [27]: enabled spatial resolution, revealed intra-tumoral heterogeneity.
**RNA in situ hybridization**	Score ≥ 2 on 0–4 RNAscope scale	Tissue sections	FGFR2 mRNA correlated with gene amplification	Kuboki 2018 [27].
**ctDNA sequencing**	Fold change ≥ 1.4 (Guardant360 panel)	Plasma samples	7.7% positive via ctDNA (14/182) vs. 2.6–4.4% by tissue. ctDNA positivity linked to poorer survival and response to FGFR inhibitors	Jogo 2021 [29], Shariff 2024 [30]: ctDNA positive patients had poorer survival; ctDNA detected heterogeneity missed in biopsy.
**CTCs (circulating tumor cells)**	≥5 FGFR2+ cells per 10 mL blood (FACS with Alexa 488 > 1000)	Blood samples	FGFR2+ CTCs correlated with worse recurrence-free survival; consistent with tissue FGFR2 status	Kuroda 2020 [28].
**NGS (next-generation sequencing)**	No numeric cutoff specified; prevalence in GC = 7.9%	Panel-based (solid tumors)	Found FGFR2 alterations in 7.9% of gastric tumors (*n* = 5557, 9.1% amplifications, 2.8% mutations, 3.1% rearrangements)	Gu 2021 [34]; denominator given, but manuscript does not provide positivity thresholds.

**Table 2:** Summary of selected anti-FGFR2 drugs.

Drug	Type	Clinical Trials/Studies (IDs)	Results	Adverse Effects	Status
**AZD4547**	Selective FGFR2 TKI	Translational trial (Pearson 2016 [66]); SHINE (NCT01457846) phase II trial vs. paclitaxel	Tumors with high-level FGFR2 amplification showed partial responses (4/9 pts) **No significant PFS benefit over paclitaxel** despite being well-tolerated	Mostly mild; well-tolerated	**Discontinued (no further dev.)**
**Futibatinib**	Irreversible FGFR1–4 TKI	Phase I (NCT02052778; JapicCTI-142552); phase II (NCT04189445)	Tumor shrinkage in 58% of pts; **ORR 17.9–26%; better response with higher FGFR2 copy numbers**	Hyperphosphatemia as main adverse effect but no maximum tolerated dose was reached	Ongoing trials (NCT05945823, phase II trial)
**KIN-3248**	Irreversible FGFR1–4 TKI	Phase I (NCT05242822)	5/54 pts partial response (9.3%); terminated early for commercial reasons	Hyperphosphatemia, diarrhea, stomatitis; 1 hypersensitivity DLT	Discontinued (trial stopped early)
**Infigratinib**	FGFR1–3 TKI	Phase II (NCT05019794)	ORR 25%; DCR 80%; tumor shrinkage in 15/19 pts (max −78.5%)	Grade 3 TRAE 42.9%, most recoverable. No drug-induced death reported	Initially given accelerated approval by the FDA for cholangiocarcinoma in 2023 but withdrawn in 2024
**Pemigatinib**	FGFR1–4 TKI	Case report (Shinomiya 2024)	Off-label use: tumor markers dropped, clinical improvement, then progression after 3 mo	Not detailed (short course)	Approved (cholangiocarcinoma); off-label in GC
**Bemarituzumab**	FGFR2b mAb	Phase I (safety, early efficacy); FIGHT (phase II, NCT03694522); FORTITUDE-101 (phase III, NCT05052801, ongoing)	FIGHT: PFS 9.5 vs. 7.4 mo; OS 19.2 vs. 13.5 mo; ORR 48.1% vs. 33% (bemarituzumab vs. control).	Manageable; improved over TKIs	Ongoing; FDA Breakthrough Therapy designation for FGFR2b positive GC (defined as >10% tumor cells staining)

## Data Availability

No new data were created or analyzed in this study.

## Abbreviations

CEN10	Centromere 10
CLDN18.2	Claudin 18.2
CTCs	Circulating tumor cells
ctDNA	Circulating tumor DNA
DISH	Dual-color in situ hybridization
DLT	Dose-limiting toxicity
EGFR	Epidermal growth factor receptor
FGF	Fibroblast growth factor
FGFR2	Fibroblast growth factor receptor 2
FISH	Fluorescence in situ hybridization
GC	Gastric cancer
HER2	Human epidermal growth factor receptor 2
IHC	Immunohistochemistry
ISH	In situ hybridization
MAPK	Mitogen-activated protein kinase
mAb	Monoclonal antibody
mFOLFOX6	Modified FOLFOX6 regimen (oxaliplatin, folinic acid, fluorouracil)
MSI	Microsatellite instability
NGS	Next-generation sequencing
ORR	Objective response rate
OS	Overall survival
PCR	Polymerase chain reaction
PD-L1	Programmed death-ligand 1
PFS	Progression-free survival
PRISMA	Preferred Reporting Items for Systematic Reviews and Meta-Analyses
RTK	Receptor tyrosine kinase
STAT	Signal transducer and activator of transcription
TMA	Tissue microarray
